# Extracellular Vesicles as Biomarkers in Cancer Immunotherapy

**DOI:** 10.3390/cancers12102825

**Published:** 2020-09-30

**Authors:** Matthen Mathew, Mariam Zade, Nadia Mezghani, Romil Patel, Yu Wang, Fatemeh Momen-Heravi

**Affiliations:** 1 Division of Hematology and Oncology at New York Presbyterian Hospital/Columbia University, Columbia University Medical Center, New York, NY 10032, USA; matthenm@gmail.com; 2 Herbert Irving Comprehensive Cancer Center, Columbia University Medical Center, New York, NY 10032, USA; 3 Cancer Biology and Immunology Laboratory, College of Dental Medicine, Columbia University Irving Medical Center, New York, NY 10032, USA; mz2616@cumc.columbia.edu (M.Z.); nm3044@cumc.columbia.edu (N.M.); 4 Division of Periodontics, Section of Oral, Diagnostic, and Rehabilitation Sciences, College of Dental Medicine, Columbia University, New York, NY 10032, USA; 5 Graduate School of Biomedical Sciences, Rowan University School of Osteopathic Medicine, Stratford, NJ 08084, USA; romilpatel119@gmail.com; 6 Department of Periodontics, University of Pennsylvania School of Dental Medicine, Philadelphia, PA 19104, USA; yuwang8@upenn.edu

**Keywords:** exosomes, extracellular vesicles, tumors, oncogenesis, immunotherapy, biomarker

## Abstract

**Simple Summary:**

Extracellular vesicles (EVs) are small particles found throughout the body. EVs are released by living cells and contain cargo representing the cell of origin. In recent years, EVs have gained attention in cancer research. Since the cargo found inside EVs can be traced back to the cell of origin, EVs shed from cancer cells, in particular, may be used to better describe and characterize a patient’s tumor. EVs have been found and isolated from a variety of bodily fluids, including blood, saliva, and amniotic fluid, and therefore offer a non-invasive way of also diagnosing and monitoring patients before, during, and after cancer immunotherapy. The aim of this review article was to summarize some of the recent work conducted in this field and the challenges we face moving forward in utilizing EVs for cancer diagnostic and therapeutic purposes in cancer immunotherapy in the clinical setting.

**Abstract:**

Extracellular vesicles (EVs), including exosomes and microvesicles, are membrane-bound vesicles secreted by most cell types during both physiologic conditions as well in response to cellular stress. EVs play an important role in intercellular communication and are emerging as key players in tumor immunology. Tumor-derived EVs (TDEs) harbor a diverse array of tumor neoantigens and contain unique molecular signature that is reflective of tumor’s underlying genetic complexity. As such they offer a glimpse into the immune tumor microenvironment (TME) and have the potential to be a novel, minimally invasive biomarker for cancer immunotherapy. Immune checkpoint inhibitors (ICI), such as anti- programmed death-1(PD-1) and its ligand (PD-L1) antibodies, have revolutionized the treatment of a wide variety of solid tumors including head and neck squamous cell carcinoma, urothelial carcinoma, melanoma, non-small cell lung cancer, and others. Typically, an invasive tissue biopsy is required both for histologic diagnosis and next-generation sequencing efforts; the latter have become more widespread in daily clinical practice. There is an unmet need for noninvasive or minimally invasive (e.g., plasma-based) biomarkers both for diagnosis and treatment monitoring. Targeted analysis of EVs in biospecimens, such as plasma and saliva could serve this purpose by potentially obviating the need for tissue sample. In this review, we describe the current challenges of biomarkers in cancer immunotherapy as well as the mechanistic role of TDEs in modulating antitumor immune response.

## 1. Background

Research in the field of extracellular vesicles (EVs) has expanded significantly in recent years, providing new insights into their biological functions, as well as their diagnostic potential. EVs are lipid enclosed membranes that are released by cells and contain compartments representative of their intracellular origin [1]. EVs are found systemically and have been isolated from several different types of biospecimens including plasma, serum, blood, saliva, and amniotic fluid [2]. Generally, EVs refer to an umbrella term encompassing various subtypes-based biogenesis pathways [3]. Microvesicles, also called shedding vesicles, range between 150 and 1000 nm in size and originate from invagination of the plasma membrane capturing cytoplasmic contents. Exosomes are smaller in size, ranging from 40–150 nm, and derived from early endosomes. Apoptotic bodies are larger, measuring 100–5000 nm, and are vesicles that originate from dying cells as they disintegrate [4,5]. EVs are associated with a wide variety of cell types and contain essential macromolecules, including DNA, microRNA (miRNA), messenger RNA (mRNA), proteins, and lipids [6].

Recent data have shown that EVs and particularly exosomes, have a tremendous impact on oncogenesis, tumor growth, signaling, and progression. They have been found to play a vital role in coordinating intercellular communication and transporting a rich array of micromolecules and signaling molecules between cancer cells and the surrounding cells that comprise the tumor microenvironment (TME) [7,8].

As immune checkpoint blockade (ICB) therapy has changed the landscape of cancer treatment; hence, a better understanding of the determinants of success and failure with this therapy is needed. Current biomarkers to assess immune response in relation to ICB are imperfect at best [9]. Accumulating evidence indicates that tumor-derived EVs (TDEs) are involved in immunological cross-talk and have the potential to be a breakthrough biomarker for cancer immunotherapy [10,11]. TDEs are particularly attractive targets as they are released at high levels from cancer cells compared to normal cells and have been isolated from a variety of biospecimens including blood, urine, cerebrospinal fluid, and saliva [12]. In this review, we address the challenges associated with the current biomarkers and also discuss the utility of TDEs for cancer diagnosis and monitoring.

## 2. Challenges with Current Biomarkers for Cancer Immunotherapy

Immune checkpoint inhibitors (ICI), such as anti- programmed death-1(PD-1) and its ligand (PD-L1) antibodies, activate an antitumor immune response through blocking inhibitory immune signaling. ICI therapy has demonstrated efficacy in various cancers including head and neck squamous cell carcinoma, melanoma, non-small cell lunch cancer, and others [13,14,15]. Unfortunately, only a subset of patients responds to checkpoint inhibitors and there is a crucial need to better understand the determinants of adaptive immune response. Various immunohistochemical and genomic biomarkers have been explored, most notably the use of tissue expression of PD-L1 and assessment of total nonsynonymous mutations harbored in tumor cells [16].

PD-L1 is present on the surface of tumor cells and, when bound to PD-1 receptors on T cells, it acts as a “second signal” (the first being antigen expressed on major histocompatibility complex (MHC) binding to T-cell receptor (TCR)) to suppress the activation of T cells. Blocking antibodies that target PD-1 and PD-L1, such as pembrolizumab (Merck) and nivolumab (Bristol Meyer-Squibb) potentiate an antitumor immune response by negating this inhibitory signal. While response rates vary by tumor type, typically a subset of patients achieved a response and even less achieved a durable response. As a biomarker, PD-L1 expression levels on tumor tissue as measured by immunohistochemistry has been demonstrated as both a prognostic biomarker as well as predictive of response to anti-PD-1 therapy [9].

Importantly, such tissue-based testing requires an adequate tissue biopsy. For certain tumors, almost one-third of patients have inadequate tumor tissue for molecular testing at diagnosis [17]. A tissue biopsy is not only invasive but can also lead to complications such as bleeding, infection, procedural complication (e.g., pneumothorax in the case of parenchymal lung biopsy). Furthermore, it may not be as accurate or practical for monitoring treatment effectiveness. Tissue biopsies also may not be fully indicative of the tumor phenotype given the heterogeneous nature of the tumors and differing levels of gene expression and metastatic potential at different sites [18]. Tissue can be biopsied from either the primary tumor or a metastatic lesion depending on a variety of factors. This, however, leads to expression levels that can vary significantly depending on where the tissue was procured. In one study, discordant PD-L1 expression levels were seen in 38% of cases between primary tumor and nodal metastases in patients with stage II and III lung adenocarcinomas [19]. This percent of PD-L1 expression by immunohistochemistry has impacted the way in which practitioners treat patients.

Immunohistochemistry staining has also been shown to have limitations as different antibodies have different sensitivities. Furthermore, the cut-off value of PD-L1 staining positivity remains a source of debate among pathologists and oncologists. There are currently four PD-L1 diagnostic assays individualized for a specific anti-PD-1/PD-L1 agent. This poses a challenge for clinicians and patients because each of these antibody clones are raised against different epitopes of the PD-L1 molecule and have different scoring systems, depending on the assay [20]. As discussed, there are several limitations to using tissue PD-L1 as a biomarker and a more predictive, non-invasive biomarker would be useful to guide the selection of patients for these therapies.

## 3. Tumor Mutational Burdens and Its Challenges

Tumor mutational burden (TMB) is the total number of non-synonymous somatic mutations of the genomic coding area which alter the amino acid sequences forming tumor neoantigens (TNAs). TNAs are created by DNA alterations that result in the formation of a novel protein sequence and are then presented on MHC molecules present on the cancer cell surface. These peptide fragments are then recognized by T cells as “non-self,” resulting in immune-mediated tumor cell death. TDEs are enriched in MHC class I and II molecules which harbor an abundance of TNAs and can stimulate host immune responses and T-cell priming in in vitro models [21].

Evidence from whole exome sequencing studies of melanoma and lung cancer suggests that response to anti-CTLA-4 and anti-PD-1 antibodies correlates with TMB [22,23]. Rizvi et al. reported that high TMB was associated with 59% higher overall response rate, a significantly longer progression-free survival, and 79% greater durable clinical benefit compared to low TMB in advanced lung cancer treated with immunotherapy [24]. Goodman et al. also observed a positive correlation between higher TMB and better outcome parameters in diverse cancers treated with immunotherapies [24].

However, using TMB as a biomarker also has its limitations. Although not all mutations result in robust immune recognition, TMB assigns an equal weight to each tumor mutation [25]. Some mutations are more readily identified as “non-self” by the immune system and are more likely to induce a stronger antitumor immune response, specifically with antigens resulting from viral open reading frames in a cancer’s genome. This is seen in patients with Merkel-cell carcinoma that have a modest TMB yet retain an extremely high treatment response rate with anti-PD-1 therapy [26].

Typically, a tissue biopsy and RNA profiling of the tumor is needed for T-cell therapies using personalized cancer-specific neoantigens. However, EV-based liquid biopsy can potentially identify expressed neoantigens, RNA landscape of tumor (mRNA, miRNAs, and other small RNAs), and DNA mutations, without the need for tissue samples. Moreover, EVs can monitor T-cell reactivity in patients who are treated by immune checkpoint blockade therapy [27].

## 4. EVs and Tumor Microenvironment

**Figure 1:**
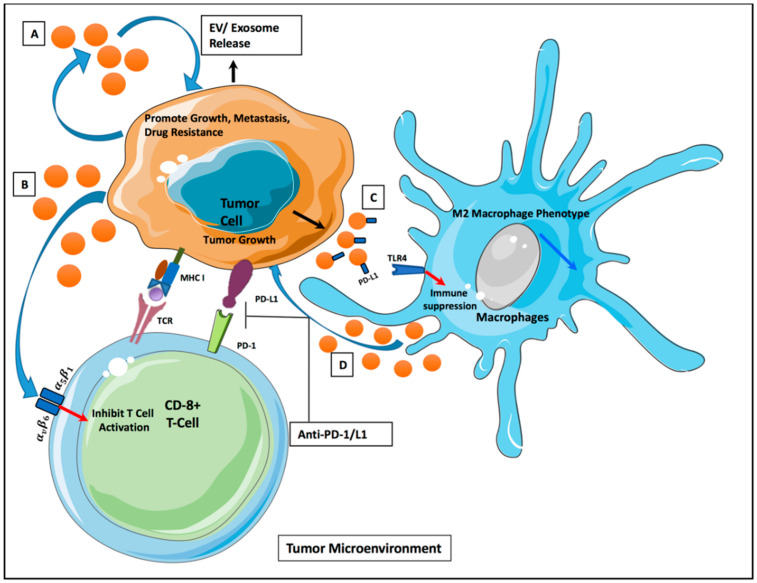
Intercellular communication via extracellular vesicles (EVs) in tumor microenvironment. (**A**) Tumor-derived EVs (TDEs) release promotes tumor growth/metastasis/progression. (**B**) TDEs release inhibits T-cell activation via alpha/beta integrin receptors. (**C**) PD-L1 expressed on TDEs leads to macrophage suppression through TLR4 signaling. (**D**) Release of EVs from macrophage promotes tumor growth via miRNA signaling.

Cancer initiation and progression is highly dependent on the nature of the surrounding microenvironment, which comprises of both cellular and non-cellular components [28]. EVs act as transport agents of communication between the cells and modulate the TME (Figure 1). A supportive stroma is essential for tumor initiation in the pre-metastatic niche. Cancer-associated fibroblasts (CAFs) are highly abundant cells found in the majority of solid tumor that have been shown to be critical in tumor initiation and progression [29]. More recently, exosomes have been found to act as a direct communicator between CAFs and tumor cells. In particular, Yao-Yin et al. showed oral squamous cell carcinoma (OSCC) cells gain a more aggressive phenotype with the acquisition of CAF-derived exosomes. Mechanistically, they found that the down regulation of miR-34a-5p in CAF-derived exosomes increased OSCC cell aggressiveness through the AKT/GSK-3β/β-catenin/Snail signaling cascade [30]. As such, EVs present as crucial communicative players capable of stabilizing the pro-tumorous environment.

In a recent study, Chen et al. demonstrated the presence of PD-L1 on melanoma derived exosomes and reported that higher levels of circulating exosomal PD-L1 negatively correlated to a poor clinical outcome after ICB [31]. Ding et al. reported that pancreatic cancer (PC)-derived EVs carry miR-212–3p, which are responsible for the inhibition of regulatory factor X-associated proteins (RFXAP), an important transcription factor for MHC-II that can lead to immune tolerance in the TME [32]. In another study, Ye et al. identified a cluster of nasopharyngeal carcinoma-derived EV-associated miRNAs (hsa-miR-891a, hsa-miR24–3p, hsa-miR-106a-5p, hsa-miR-1908, and hsa-miR-20a-5p) that collectively downregulate the MAPK1 and JAK/STAT signaling pathways in T cells, thereby contributing to immune suppression [33].

The complex puzzle behind organ-specific metastasis has only just begun to be decoded in recent years. EVs have been studied as potential players in organotropism. The factors released by tumor exosomes are central in mechanisms leading to pre-metastatic niche formation such as vascular leakiness and immune cell recruitment [34,35]. A hallmark study conducted by Lyden group showed evidence of TDEs being capable of influencing specific cellular changes in future tumorous sites in a non-random manner [34]. The group described this discovery as “cell-type specificity of exosomal education.” As such, exosomes seem to be capable of not only mediating tumor growth once it has been initiated, but also directing future metastasis in a tissue-specific manner.

## 5. EVs as Cancer Biomarkers

In 1998, the National Institute of Health defined a biomarker as “a characteristic that is objectively measured and evaluated as an indicator of normal biological processes, pathogenic processes, or pharmacologic responses to a therapeutic intervention” [36]. EVs fit into all three of these categories; they regularly participate in intercellular communication under normal processes, have repeatedly shown to be both qualitatively and quantitatively unique in diseased states such as cancer, and have shown to play a significant part in drug resistance. Additionally, EVs have shown to be differential and unique in diseased-states [37,38,39]. In fact, using EVs as a biomarker uses the principle of “less being more” by reducing biological sample complexity.

Inherit characteristics of EVs make them ideal candidates for aiding cancer diagnosis and prognosis, as well as monitoring therapeutic response. The ability to circulate through various bodily fluids allows them to have the advantage of being a non-invasive testing option. In addition, EVs offer a stable vehicle for genomic testing as their lipid bilayer protects biomacromolecules, such as RNA and proteins, from enzymatic activity. The increased level of EVs and the contrast between tumor cell, EV proteome, and nucleic acid cargos reflects the potential of using EVs for early diagnosis and monitoring of cancer [40].

Preliminarily studies have shown plasma EV profiling offers a highly sensitive method of tumor analysis [41]. An early study comparing the content of glioblastoma EVs to their cells of origin found that the TDEs were snapshots of the content from the secreting cell [42]. The presence of known cancer-associated miRNA, mRNA, long non-coding RNA(IncRNA), and post translational protein modifications in TDEs have since been established in multiple cancer types [43]. In a study on ovarian cancer biomarkers, it was demonstrated that malignancies could be reliably distinguished from benign disease based on the levels of eight specific exosomal miRNAs [44]. EVs isolated from the circulation of patients with head and neck cancers carry oncogenic miRNA signatures, tumor-derived DNA fragments, tumor-specific protein, and RNA signatures [12]. Additionally, a study conducted by Thakur et al. has shown that exosomal DNA (exoDNA) represents the whole genomic DNA and reflects the mutational status of parental tumor cells [45].

Although circulating free DNA (cfDNA) can also provide a non-invasive liquid biopsy method, exosomes are found to be more advantageous. In mutation detection of early stage, non-small cell lung cancer, exosomes were found to be more sensitive (25.7% compared to 14.2%, respectively) and more specific (96.6% and 91.7%, respectively) than cfDNA [46]. Furthermore, a study by Allenson et al. found that a higher percentage of patients with early stage pancreatic cancer exhibited detectable KRAS mutations in exoDNA than previously reported for cfDNA [47]. There is also evidence that cfDNA reflects the nucleic acid complement of dying or apoptotic cells rather than viable solid cells [48].

The increased production and secretion of EVs by tumor cells allows them to be a useful tool in cancer detection and disease progression [49]. Research has shown elevated amounts of EVs in certain disease states and in several types of cancers including, glioblastoma, ovarian cancer, melanoma, renal cancer, and oral squamous carcinoma [50,51,52,53,54]. Several distinct mechanisms may be involved in the increase of EV secretion by tumor cells. Previous literature has suggested that hypoxia and stress in the TME are factors that can account for this abundant EV secretion by tumor cells [55]. EV production and release was also reported to be regulated by the p53 protein, Heparanase, and the Rap GTPase proteins [56].

It has also been suggested that early tumor-EV release may provide opportunities for early disease detection. In an in vivo model of pancreatic cancer, the level of EV-associated protein marker was increased prior to the tumor being detectable by imaging modalities [57]. EVs showed an active role in mediating resistance to different therapies by mediating biomacromolecule transfers. In a series of studies in colon cancer, breast cancer, and soft tissue sarcoma, exposure to EVs derived from drug resistant cells was able to disrupt drug-associated signaling in sensitive recipient cells and contribute to the tumor resistance [58,59,60,61,62]. In conclusion, these observations have suggested that tumor EV biomarkers have potential prognostic and predictive value.

## 6. EVs and Cancer Immunotherapy

TDEs can carry immunosuppressive molecules such as PD-L1, TGFβ1, FasL, TRAIL, and NKG2D ligands which make them important mediators of tumor immune evasion and possible targets for immunotherapy [63,64,65,66,67]. Metastatic melanoma-derived exosomes, which are stimulated by interferon-γ (IFN-γ), expressed more PD-L1 on these vesicles and inhibited antitumor responses [31]. When expressed on the tumor cell surface, PD-L1 facilitates evasion of immune surveillance by interacting with programmed death-1 (PD-1), thereby inhibiting T-cell function. Metastatic melanomas release EVs that carry PD-L1 and suppress the cytotoxic function of CD8^+^ T cells [31]. The EV-associated PD-L1 demonstrate the same membrane topology as cell surface PD-L1 and suppress T cells in similar manner [31]. PD-L1-expressing EVs were isolated from human blood and their levels of PD-L1 expressed on the EVs, not the soluble form of PD-L1, was associated with disease progression in head and neck cancer [68]. Consistently, in the 4-nitroquinoline 1-oxide-induced malignant oral/esophageal injury model, EVs carrying PD-L1 isolated from supernatants of murine or human HNSCC cell lines hampered the infiltration of CD4^+^ T and CD8^+^ T cells into the tumor microenvironment, thereby accelerating tumor progression [69]. In patients with melanoma, TDE PD-L1 is also a marker of immune activation early after initiation of therapy with PD1-targeting antibodies and predicts a clinical response to PD1 blockade [70].

In addition to PD-L1, other immunosuppressive molecules such as TGFB1 and NKG2D ligands were also enriched in TDEs and were able to induce T-cell suppression [71]. Both TGF-β and/or ligands for the activating receptor NKG2D have been described to negatively affect NK cell functions [72]. NKG2D is an activating receptor expressed by NK cells and some subsets of T cells and represents a major recognition receptor for detection and elimination of cancer cells. The NKG2D ligands are stress-induced self-proteins that can be secreted as soluble molecules by protease-mediated cleavage. The release of NKG2D ligands in the extracellular milieu is considered a mode of finely controlling their surface expression levels and represents a relevant immune evasion mechanism employed by cancer cells to elude NKG2D-mediated immune surveillance [73].

TDE expressing NKG2D ligands are a means that tumor cells use for immune-evasion [74]. It has been demonstrated that ovarian cancer and melanoma TDEs, express NKG2D ligands and prevent activation of cytotoxic NK cells [75,76]. Expression of FASL and TRAIL on TDEs induce apoptosis in dendritic cells (DCs) and peripheral blood mononuclear cells (PBMCs) which causes immunosuppression and promotes tumor progression [77]. TDE bearing FASL eliminate antigen-specific effector T cells. Other inhibitory, immunosuppressive proteins—such as FASL, TGFβ1, TRAIL, COX2, CD39/CD73, PDL1, CTLA4, and others—are reported to be associated with TDEs [78].

Besides immunosuppressive role of TDEs that could be blocked for better immunotherapy outcomes, TDEs could be used to activate the immune system. For example, DCs act in the first step of the cancer immunity cycle, eliminating tumor cells through T-cell activation [79]. As they share surface membrane components that interact with other immune cells, DC-derived EVs can potentially be engineered to act as cell-free antitumor vaccines, providing a novel immunotherapy in the fight against cancer [80]. Particularly, EVs released by DCs contain major histocompatibility complex (MHC) class I and class II molecules that are able to activate cognate T cells and promote humoral responses. These activities motivated the use of DC-derived EVs in the treatment of cancer, infectious diseases, and autoimmune disorders [81]. These findings indicate that TDEs play a major role in tumor immune evasion and growth. Beside their biomarker utility, TDEs could be targeted or engineered for cancer treatment. In fact, TDEs surface molecules could be manipulated and provide cancer treatment. In this way, tumor-associated antigens, immunogenic peptide, and other molecules such as heat shock proteins could be engineered in the future for cancer treatment.

## 7. Challenges for Using EVs as Biomarkers: ISOLATION, Clinical Challenges, and EV Heterogeneity

### 7.1. Challenges in EV Isolation and Assay Development

It is known that the EV subpopulations obtained by different isolation methods can substantially differ and this can considerably influence the EV-based bioassay results [82]. The traditional methods used for EV isolation such as ultracentrifugation and filtration utilize the EV physical properties, such as size and buoyant density [1]. Precipitation methods using polyethylene glycol [83] and other commercial precipitation reagents are another means of EV isolation [1]. In addition to those methods, numerous novel technologies for the isolation of the EV population based on specific interactions with the EV surface molecules or microfluidic technologies have recently been developed [84,85]. Each of these methods has its specific advantages and disadvantages, which have to be taken into consideration when planning an experiment in EV biomarker discovery. These methods along with their advantages and disadvantages are listed in Table 1. In using EVs for cancer biomarker discovery, recovering reproducible population of EVs in biofluids requires a substantial amount of vesicles/biological material. A desired method for the isolation of EVs for biomarker discovery should be relatively inexpensive, simple, and fast, and allow for the isolation of EVs from a large number of samples.

Traditional methods of EV isolation including ultracentrifugation, precipitation methods, and filtrations do not discriminate between different types of vesicles. As a result, a mixed population of vesicles is obtained in most studies of EVs’ signature which makes the comparisons difficult [99]. This disadvantage can mask some of the molecules with high specificity and sensitivity that are associated with a specific EV subpopulation. The standardization of protocols for isolation methods is necessary to be able to reproduce the results and to avoid any interference with verification, reproducibility, and combining data from various research teams.

In designing any cancer biomarker study, it is crucial to comprehensively study and standardize the protocols for EV isolation. Arrays of complementary methods such as nanoparticle tracking analysis, transmission electron microscopy, immunohistochemical methods, dynamic light scattering, and proteomic characterization of EV subtype should be used to characterize and standardize the EV morphology. In addition, pre-analysis protocols such as sample storage and viscosity adjustment should be taken into consideration to increase reproducibility and isolation efficacy.

Besides difficulties with isolation there are challenges in accurate measurement of cargoes after isolation. Some of the detection methods may not be accurate due to the lower abundance of cargo and absence of ribosomal RNA in EVs [100,101]. Moreover, the choice of assay can change the sensitivity of the results; for instance, there is a tradeoff between using microarray characterization of miRNA in EVs with lower cost versus RNA sequencing with higher cost and broader dynamic range [102]. In fact, using high accuracy platforms such as digital droplet PCR showed increased assay accuracy in the analysis of EV-based nucleic acids [103]. Vortexing the biofluid after thawing increased the EV recovery [104]. In addition to the challenge of a low concentration of nucleic acids in EVs, determination of endogenous miRNA for qRT-PCR normalization in biospecimens should be considered [105]. As scientific methodology gears toward more thorough reporting of EV handling and measurement protocols, the studies’ reproducibility and compatibility will improve. Finally, the most appropriate EV isolation methods will depend on the sample type, the purpose of the study, the downstream analyses, target biomacromolecules and the available equipment and resources. Reproducible standard operating procedures for sample handling and isolation techniques will lead to maximum yield and quality.

Altogether, it might neither be desirable nor possible to develop a universal method for EV isolation for all cancer biomarker studies; however, standard methods should be developed to solve particular types of problems in biomarker discovery. Standardization and development of reproducible and applicable methods for EV isolation aimed at applying them in cancer biomarker discovery and clinical studies is a high priority task in the field.

### 7.2. Clinical Challenges and Variables for Standardization of EV-Based Biomarker Discovery

Besides challenges in isolation, handling, and analysis, there are several other issues to be addressed before the potential use of EVs as cancer biomarkers. For example, the level of circulating EVs has been shown to be affected by different factors including the time of sample collection [106], physical activity [107], gender [108], and ethnicity [109]. These factors, if not accounted for, may influence the subsequent analysis and conclusions. Collection, isolation, and storage protocols for biospecimens for EV biomarker discovery should be thoroughly optimized and standardized. For example, storage of EVs at −80 °C leads to complete recovery of EVs compared to the fresh urine in contrast to storing at −20 °C which leads to a decrease in EV recovery [104]. The feasibility of high throughput isolation of analyses and isolation of tumor-derived EVs from complex biological fluids are yet to be demonstrated.

Another clinical challenge of EV-based biomarker discovery is EV heterogeneity and change in different stages of disease which necessitate close monitoring and characterization of the study cohort. For instance, the miRNA signature of a tumor is changing when metastasis occurs [110]. Moreover, the reproducibility of EV-associated tumor signatures in independent cohorts is needed. During the clinical validation processes, tumor signatures in EV biomarkers should be evaluated as to whether the signature is correlated with other valid non-cancer biomarkers and clinical outcomes such as tumor burden, metastasis, mutational burden. Finally, for the clinical application of EV biomarkers, valid methods for the quantification of EV biomarkers and evidence of target engagement in a clinical trial are necessary. Therefore, the development of valid EV-based cancer biomarkers will take a long time and need significant optimization and validation in clinical settings. Current challenges in the development of EV-based cancer biomarkers and suggested strategies to overcome the challenges are summarized in Table 2.

### 7.3. EVs Heterogeneity

Extracellular vesicles (EVs) can be classified in the three categories of exosomes, microvesicles, and apoptotic bodies based on the mode of biogenesis. Consistent with different underlying biogenesis mechanisms, cells release multiple populations of EVs with different functions. For instance, enrichment of the packaging of specific miRNAs in exosome fraction has been documented [54,55,111].

Historically, lack of guidelines regarding the nomenclature of EVs, limitations with early characterization and isolation methods resulted in a mix nomenclature and study of overlapping populations [112]. Recently, new technologies and proteomic characterization of EVs could help us to classify EVs based on biological origin and characteristics, and enhance our knowledge of EV subpopulation and specific content. This heterogeneity is one of the challenges in the field of EV biomarker discovery. By using standardized operating protocols, new technologies for isolation and molecular profiling of EV, we will better understand the role of EVs in physiological and pathological conditions with a higher resolution. These advances can pave way to translation of these findings to diagnostic and therapeutic realms.

The term “oncosome” is often used synergistically with “TDE.” The term was originally adopted to specifically describe TDEs with oncogenic material shed by glioma cells [113]. The extreme variation in the size of TDEs/oncosomes subsequently led to the more recently adoption of the term “large oncosome” (LO) [114]. LOs have been described as unusually large tumor-derived exosomes, ranging from 1–10 um in diameter [115]. Although often more than 1000 times larger than the traditional TDE/oncosome, LOs have been shown to be unique beyond their physical dimension [115,116]. LOs have been associated with more-rapid metastasis and miRNA profiling has shown differential miRNA content in larger oncosomes compared to the traditionally sized TDE [115,117]. More specifically, LOs are shed from tumor cells with an amoeboid phenotype, which is characteristic of highly aggressive cancers [117]. Therefore, the molecular content found in LOs may be more specific to metastasizing cancer cells when compared to regular size TDEs shed from the same cell of origin. However, more studies are needed to more clearly outline the differences between LOs and TDEs regarding protein cargo and target cell effects before applying LOs to cancer monitoring. This novel subset of TDEs is yet another reminder for investigators to carefully define and characterize the EV in question, not just for themselves but for the scientific community and application of LOs in the clinical setting.

## 8. EVs’ RNA, DNA, Proteomics for Cancer Biomarker Discovery

Although ICI, such as anti-CTLA4, anti-PD-1/PD-L1, and other molecular-targeted therapies have advanced cancer therapy in recent years, reliable and sensitive protocols for tumor monitoring during such treatments are currently lacking [37]. Traditional tumor biopsies are generally nonspecific, invasive, and costly. Furthermore, tissue biopsies fail to provide a complete picture of the intricate heterogeneity that exists within the tumor and its microenvironment, most notably during metastasis and therapy. As such, there is an urgent need to better investigate and characterize non-invasive and tumor-specific biomarkers for early diagnosis, monitoring tumor growth, evaluating prognosis, and predicting immunotherapy response.

The biochemical nature and origin of EVs present these nanoparticles as promising biomarkers in cancer diagnosis, prognosis, and treatment monitoring. EVs’ cargo has been previously shown to resemble the molecular profile of the parent tumor cell as well as promote pro-tumorigenic events such as endothelial cell migration, angiogenesis, immunosuppression, and multidrug resistance [118,119]. EVs’ RNA, DNA, and proteins are protected by the natural lipid bilayer capsule from adverse biological (e.g., RNAses, DNases, proteases) and environmental conditions [97,120,121,122,123,124]. Additionally, EV signatures can be detected from a number of human body fluids, including serum, plasma, saliva, and urine. Not only do whole EV levels increase in tumor states, but EV markers and cargos have also shown to be specific and/or upregulated in certain tumors.

EV-mediated transfer of miRNAs has been shown to play essential functions in protein phosphorylation, RNA splicing, and immune system modulation in the surrounding microenvironment [120,122]. There is significant evidence that cellular RNA is selectively loaded into EVs before their release into the extracellular environment [97,122,125]. EVs have also been well described as being capable of regulating gene networks as well as being able to drive differential cell phenotypes on the receiving cell through such RNA transfer [126]. Although many EV signatures have been documented in different cancer types, the significance of such signatures and correlation with clinical findings must be further investigated.

Lin et al. recently explored the clinical utility of salivary EV RNA in esophageal cancer. The group found that levels of a particular salivary EV chimeric RNA (GOLM1-NAA35) reflect tumor burden in vivo [127]. Furthermore, it appears to be accurate in longitudinal monitoring of treatment response. A number of other studies have indicated that specific mRNA species show differential expression in responsive and unresponsive patients receiving immunotherapy [128,129,130]. Liu et al. evaluated the prognostic and predictive value of serum EV-associated miRNAs for tumor recurrence and response to therapy in colon cancer. They were able to identify 145 differentially expressed miRNAs. After more carefully validating the top results, they found that miR-4772-30 under-expression was significantly related to increased risk of tumor recurrence and risk of death [131].

Another recent study showed that exosomal CAFs are intrinsically resistant to cisplatin and play a functional role in head and neck cancers through the delivery of miR-196a to tumor cells [132]. It was found that miR-196a-depleted exosomes from CAFs actually restored cisplatin sensitivity in head and neck cancers. Each of these novel findings present themselves as possible therapeutic targets in blocking the essential physical and physiological nature of the tumor cells’ microenvironment. As more studies reveal the particular EVs’ cargo and quantitative threshold specific for cancer types, subtypes, or stages, their use in precision medicine has the potential to change the face of cancer therapy.

TDEs also show predictive value in tumor recurrence during therapy [38]. A recent study conducted by Theodoraki et al. serially monitored for TDEs in a group of head and neck cancer patients receiving a combination of cetuximab, ipilimumab, and radiation therapy. The group evaluated the predictive potential of the EV molecular cargo for disease recurrence and found that the TDEs to total exosome ratio increased from baseline levels. It was concluded that TDEs can be used to effectively monitor patient response during immunotherapy and can also be used to direct appropriate treatment plans [133].

Circulating exosomal double-stranded DNA (dsDNA) reflects the mutational status of the paired tumor DNA [134]. In one study, serum-derived EVs of patients with neuroendocrine tumors were shown to contain dsDNA that covered all chromosomes. In patients with pancreatic cancer, previously identified mutant KRAS and TP53 DNA were also detected in circulating EVs [47,135]. Once better described, EV dsDNA could potentially be used as a noninvasive genetic tool for mutation detection in treatment planning and assessment prior to surgery. EV proteomics have also been explored in the context of tumor activity. Specific proteomic signatures both on and released by EVs appear to drive suppression of host immune cells, furthering the growth and survival of the tumor cells [38,119]. In the serum of non-small cell lung cancer patients, EV lncRNA MALAT-1 was shown to be positively associated with tumor stage and lymphatic metastasis. In addition, in vitro studies showed that this RNA signature promotes tumor growth and migration [136].

Such studies reflect the promise of EVs’ RNA, DNA, and proteomics not only in cancer screening or diagnosis, but also as a noninvasive monitoring tool in patients receiving immunotherapy. TDEs have been shown to contain RNA signatures that are specific and sensitive in a subset of cancer types; RNA signatures appear to be down-regulated or up-regulated in some tumorous environments compared to others. EVs appear to be capable of influencing cell activity at future sights of metastasis. Such findings shed light on the predictive utility of EV markers in the clinical setting. The proposed indication of EV signatures in different cancer types is summarized in Table 3.

## 9. EVs for Cancer Therapy

The potential use of EVs in cancer therapy has been reviewed in depth [149,150]. EVs naturally carry biomacromolecules—including different RNAs (mRNAs, regulatory miRNAs), DNAs, lipids, and proteins—and can efficiently deliver their cargoes to recipient cells, eliciting functions, and mediating cellular communications [151]. EVs confer some advantages for drug delivery: (1) EVs are small and have a high efficiency for delivery because of their similarity to cell membranes [152]. (2) EVs are biocompatible, non-immunogenic, and non-toxic, even in repeated in vivo injections [97,153]. (3) EVs are stable even after several freeze and thaw cycles, and their lipid bilayer protects the protein and RNA cargoes from enzymes such as protases and RNases. (4) EVs have slightly negative zeta potential, leading to long circulation [154]. (5) EVs also exhibit an increased capacity to escape degradation or clearance by the immune system [155].

In addition to endogenous ability of EVs for therapy, introduction of exogenous nucleic acids has been used for the delivery of RNA interference (RNAi), miRNAs, and mRNA [88,97,156] and can be tailored to modify the tumor microenvironment. Tumor cell antigens and immune stimulatory molecules on TDEs can be used for immune cell priming [157,158]. Different immune-stimulatory molecules can also be introduced to EVs to modulate anti-tumor immunity and act as EV-based tumor vaccines [159]. Tumor cells producing the EVs can be engineered to express specific cytokines/chemokines that have an immunomodulating effect on the tumor microenvironment [159,160].

Taken together, EVs provide a special biological tool of targeting tumors and their microenvironments that can potentially increase the efficacy and minimize cytotoxic and immunogenic side effects associated with conventional therapies. However, several limitations still have to be overcome. For instance, the isolation methods should be optimized to enable the isolation of a specific subpopulation of EVs with desirable cargoes. The processes for loading of EVs should be optimized for better efficiency, and the whole pipeline of standard operating procedure for EV isolation, characterization, and modification/loading should be developed. The heterogeneity of EVs should be addressed, and the bioavailability and biodistribution of EVs should be characterized in in vivo studies.

## 10. Conclusions and Future Directions

TDEs play critical mechanistic roles in TME and modulate oncogenic pathways in cancer cells in order to promote tumor progression, metastasis, and therapy resistance. As previously mentioned, qualitative and quantitative findings of specific EV molecular signatures are necessary in applying EV-based tumor diagnosis, monitoring, and therapeutic delivery to specific cancer subtypes. Studies are beginning to shift their focus from total-EV research in healthy versus disease state toward more specific EV molecular cargo, as described above. Although a great amount of future work on this topic remains necessary, EVs have the potential to be a promising biomarker and therapeutic tool for cancer treatment planning and monitoring in the era of precision medicine.

## Figures and Tables

**Table 1:** Methods for isolation of EVs.

Isolation Methods	Time	Indication	Advantages	Disadvantages	References
Ultracentrifugation/differential centrifugation	3 h–12 h	Large volume of biofluids	Most commonly used, could be combined with other methods such as size exclusion, immune affinity isolation and sucrose gradient method	Need of expensive equipment, time consuming, low efficiency, deformity, impurity and protein co-aggregation, limit in processing sample quantity, low RNA yield	[86,87,88,89]
Size exclusion (filtration+ chromatography)	2 h	Large volume of biofluids, could be combined with nano-membrane ultrafiltration concentrators	Feasible, quick, inexpensive, low risk of contamination/deformity, yields functional EVs	EV dilution, yield variation	[90,91,92,93]
Immune affinity isolation (antibody against EVs surface proteins)	4–6 h	High purity isolation of EVs, isolation of sub-set of EVs, isolation of EVs from viruses and LPP	High specificity and selectivity, reproducibility, isolating special sub-set of EVs and possibility of negative selection	Cross reactivity of antibody, costly, low yield, expensive equipment	[94,95] (Bukong et al., 2014, Momen-Heravi et al., 2013)
Microfluidic technologies	5–14 µL/min	Low volume of input biofluids	Can be combined by immune affinity methods	Early stage of development, low throughput, high cost	[96]
Participation with hydrophilic polymers	1 h (some protocols overnight)		Relatively low cost and high yield of EVs and biomolecules, simplicity, no need for expensive equipment	Contamination of EVs with protein complexes and lipoproteins, polymer retention	[97]
Porous structures (Capturing EVs through in ciliated micropillar structure)	2 h	Selectively trap particles in the range of 40–100 nm based on the research question	Purity, rapidness	Not suitable for isolation of larger particles, not validated with clinical samples, can handle only small amount of biofluids	[96,98]

**Table 2:** Current challenges in the development of EV-based cancer biomarkers and suggested strategies to overcome the challenges.

Developmental Process	Current or Future Challenges	Required Strategies
Sample processing and pre-analytical steps	Presence of different isolation methods and protocols Limitation in availability of standardized protocols Variability in sample collection, storage, and handling	Standardization of isolation methods, storage, and samples handling Development of novel methods for preparing EVs with high purity and yield
Biomarker discovery	Use of candidate approach and lack of comprehensive Omics study for EV characterization in cancer research Lack of well-characterized cohort and access to the clinical/demographic status of patients in EV biomarker studies Variability in utilized technologies (e.g., qPCR versus next generation sequencing)	Using unbiased approaches to identify candidate biomarkers Establishment of well-characterized cohort Optimization and standardization of assays Correlating EV signature with tumor molecular characteristics and known cancer biomarkers
Clinical validation	Need for independent large well-characterized cohort studies to assess the biomarker utility of EVs Need for longitudinal sampling to monitor disease progression and correlate EV signature with clinical outcome	Evaluation of EVs as cancer biomarkers in independent cohorts Correlating tumor EV biomarkers with clinical presentations, disease free survival, and disease progression
Clinical feasibility	Need for development of actionable panel of EV markers	Develop relatively inexpensive, rapid assays with documented clinical utility

**Table 3:** Proposed indication of EV signatures from human body fluids in different cancer types.

Class	Exosome Biomarker	Cancer Type	Biofluid	Indication	References
RNA	MEG3	Bladder	Serum	Diagnosis, recurrence	[137]
	HOTAIR	Bladder	Urine	Prognosis	[138]
	miR-21	Esophageal	Serum	Diagnosis, prognosis	[139]
	miR-4772-3p	Colon	Serum	Recurrence	[131]
	PCA-3, TMPRSS2:ERG	Prostate	Urine	Diagnosis, monitoring	[140]
	MALAT-1	Lung	Serum	Diagnosis, prognosis	[136]
	GOLM1-NAA35	Esophageal	Saliva	Early detection, recurrence, therapeutic response	[127]
	miR-141, miR-375	Prostate	Serum	Diagnosis, prognosis	[141]
DNA	*RET*, *HIF2A*, *VHL*, *SDHB*	Neuroendocrine	Serum	Genetic diagnosis	[134]
	*KRAS^G12D^*, *TP53^R273H^*	Pancreatic	Serum	Genetic diagnosis	[47,135]
Proteomics	CD36, CD44, 5T4, basigin, CD73	Bladder	Urine	Diagnosis	[142]
	LRG1	Lung	Urine	Diagnosis	[143]
	BARF1	Nasopharyngeal	Serum, saliva	Diagnosis	[144]
	CD24, EpCAM, TGF-B1, MAGE3/6	Ovarian	Plasma, ascitic	Diagnosis, prognosis, therapeutic response	[145,146]
	Fibronectin	Breast	Plasma	Early detection	[147]
	GPC1	Pancreatic	Serum	Diagnosis, prognosis	[57]
	MMP9	Renal	Urine	Diagnosis	[148]
